# Analgesic and Anti-Inflammatory Activities of Salicylaldehyde 2-Chlorobenzoyl Hydrazone (H_2_LASSBio-466), Salicylaldehyde 4-Chlorobenzoyl Hydrazone (H_2_LASSBio-1064) and Their Zinc(II) Complexes

**DOI:** 10.3390/molecules16086902

**Published:** 2011-08-15

**Authors:** Walfrido Bispo Júnior, Magna S. Alexandre-Moreira, Marina A. Alves, Anayive Perez-Rebolledo, Gabrieli L. Parrilha, Eduardo E. Castellano, Oscar E. Piro, Eliezer J. Barreiro, Lídia Moreira Lima, Heloisa Beraldo

**Affiliations:** 1 LaFI Laboratório de Farmacologia e Imunidade, Instituto de Ciências Biológicas e da Saúde, Universidade Federal de Alagoas, Maceió, AL, Brazil; 2 LASSBio Laboratório de Avaliação e Síntese de Substâncias Bioativas (LASSBio, http://www.farmacia.ufrj.br/lassbio/), Faculdade de Farmácia, Universidade Federal do Rio de Janeiro, P. O. Box 68024, 21944-971, Rio de Janeiro, RJ, Brazil; 3 Departamento de Química, Universidade Federal de Minas Gerais, 31270-901, Belo Horizonte, Brazil; 4 Instituto de Física de São Carlos, Universidade de São Paulo, 13560-970, São Carlos, SP, Brazil; 5 Departamento de Física, Facultad de Ciencias Exactas, Universidad Nacional de La Plata and Instituto IFLP (CONICET – CCT La Plata), C.C. 67, 1900 La Plata, Argentina

**Keywords:** acylhydrazones, zinc(II) complexes, analgesic activity, anti-inflammatory activity

## Abstract

Salicylaldehyde 2-chlorobenzoyl hydrazone (H_2_LASSBio-466), salicylaldehyde 4-chlorobenzoyl hydrazone (H_2_LASSBio-1064) and their complexes [Zn(LASSBio-466)H_2_O]_2_ (**1**) and [Zn(HLASSBio-1064)Cl]_2_ (**2**) were evaluated in animal models of peripheral and central nociception, and acute inflammation. All studied compounds significantly inhibited acetic acid-induced writhing response. Upon coordination the anti-nociceptive activity was favored in the complex **1**. H_2_LASSBio-466 inhibited only the first phase of the formalin test, while **1** was active in the second phase, like indomethacin, indicating its ability to inhibit nociception associated with the inflammatory response. Hence coordination to zinc(II) altered the pharmacological profile of H_2_LASSBio-466. H_2_LASSBio-1064 inhibited both phases but this effect was not improved by coordination. The studied compounds did not increase the latency of response in the hot plate model, indicating their lack of central anti-nociceptive activity. All compounds showed levels of inhibition of zymosan-induced peritonitis comparable or superior to indomethacin, indicating an expressive anti-inflammatory profile.

## 1. Introduction

Inflammation is a response of the immune system to physical and/or chemical and/or biological injury, understanding by injury any process able to cause tissue or cellular damages. The types of inflammation (*i.e*. acute or chronic) differ by cause, mechanism, outcome, and intensity. It is well known that while acute inflammation has a physiological role in normal circumstances, chronic inflammation exerts detrimental effects on the functional status of cells and tissues. As a consequence inflammatory processes take part in a huge number of diseases such as atherosclerosis, Alzheimer disease, Parkinson disease, cancer, asthma, arthritis and so on [1,2,3].

Zinc is one of the most prevalent trace elements in the human body. It has been shown to be essential to the structure and function of a large number of macromolecules and for a variety of enzymatic reactions, which mediate a wide range of physiological processes [4,5]. These include production of collagen and other extracellular matrix proteins, modulation of immunoregulatory (e.g., T and B lymphocytes, macrophages, and antigen-presenting dendritic cells) and inflammatory (e.g., eosinophils, neutrophils, and mast cells) cells function. As a consequence, zinc may be considered an important immunoregulatory agent with anti-apoptotic and anti-inflammatory activities [6].

**Figure 1:**
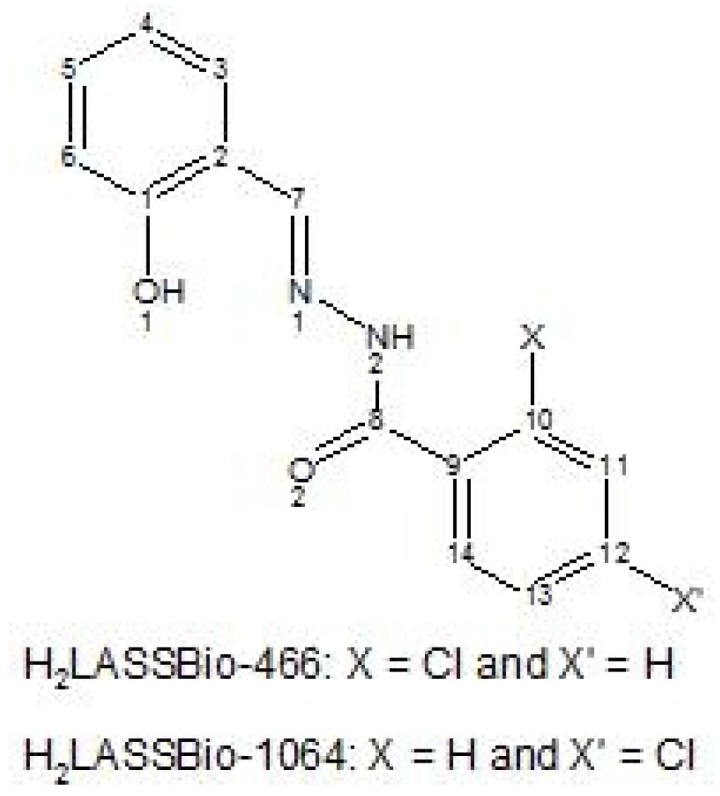
Generic structure of salicylaldehyde 2-chlorobenzoyl hydrazone (H_2_LASSBio-466) and its regioisomer salicylaldehyde 4-chlorobenzoyl hydrazone (H_2_LASSBio-1064).

In the context of a research program that aims to contribute to the discovery of new anti-inflammatory and analgesic drug candidates, we describe the synthesis of zinc(II) complexes with salicylaldehyde 2-chlorobenzoyl hydrazone (H_2_LASSBio-466) and its regioisomer salicylaldehyde 4-chlorobenzoyl hydrazone (H_2_LASSBio-1064) (Figure 1), together with a pharmacological evaluation of all acylhydrazones and zinc(II) complexes in animal models of peripheral and central nociception and acute inflammation.

## 2. Results and Discussion

### 2.1. Formation of the Zinc(II) Complexes 1 and ***2***

Microanalyses and molar conductivity data are compatible with the formation of [Zn(LASSBio-466)H_2_O] and [Zn(HLASSBio-1064)Cl]. In the first compound a di-anionic acylhydrazone is attached to the metal center upon deprotonation at N(2)-H and O(1)-H. The remaining coordination site is occupied by a water molecule, as indicated by the infrared spectrum of the compound (Section 2.3). In the second, a mono-anionic acylhydrazone is attached to the metal upon deprotonation at O(1)-H, together with a chloride ion.

Upon recrystallization of [Zn(HLASSBio-1064)Cl] in 1:9 DMSO-acetone, crystals of [Zn_2_(LASSBio-1064)_2_(H_2_O)_2_]∙[Zn_2_(LASSBio-1064)_2_(DMSO)_4_] were obtained. The compound consists of two center symmetric binuclear zinc(II) complexes hosted in the same lattice, as previously reported by some of us [7]. We had also obtained [Zn(LASSBio-1064)]_2_, which is a phenoxo-bridged dimer since the monomer would contain zinc(II) with coordination number three, which is very unlikely [7]. Hence [Zn(HLASSBio-1064)Cl] most probably also exists as a dimer, [Zn(HLASSBio-1064)Cl]_2_.

We now obtained [Zn(LASSBio-466)(H_2_O)], in which a water molecule occupies the fourth coordination position, like in the first center symmetric unit of [Zn_2_(LASSBio-1064)_2_(H_2_O)_2_]∙[Zn_2_(LASSBio-1064)_2_(DMSO)_4_]. Therefore, [Zn(LASSBio-466)(H_2_O)] is also probably a phenoxo-bridged dimer, [Zn(LASSBio-466)(H_2_O)]_2_. Considering that dimerization is favored in this class of compounds [7], the zinc(II) complexes are hereafter formulated as [Zn(LASSBio-466)(H_2_O)]_2_ (**1**) and [Zn(HLASSBio-1064)Cl]_2_ (**2**).

**Figure 2:**
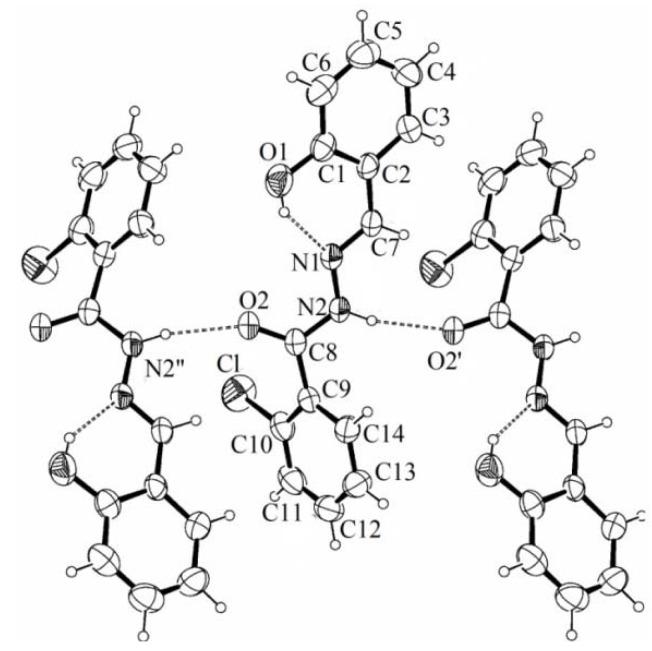
Molecular plot of LASSBio-466 showing the labeling scheme of the non-H atoms and their displacement ellipsoids at the 50% probability level.

### 2.2. Crystal Structure of H_2_LASSBio-466

Crystal data and refinement results are summarized in Table 1. Figure 2 is an ORTEP [8] drawing of the molecule and Table 2 shows the corresponding intra-molecular bond distances and angles. 

**Table 1:** Crystal data and structure solution methods and refinement results for salicylaldehyde 2-chlorobenzoyl hydrazone (H_2_LASSBio-466).

Compound		H_2_LASSBio-466
Empirical Formula		C_14_H_11_ClN_2_O_2_
Formula Weight		274.70
Temperature, K		294(2)
Crystal System		Monoclinic
Space Group		P2_1_/c
Unit cell dimensions	a, Å	9.889(1)
	b, Å	13.360(2)
	c, Å	10.079(1)
	α, °	90
	β, °	93.10(1)
	γ, °	90
Volume, Å^3^		1329.7(3)
Z, Density calc., Mg/m^3^		4, 1.372
Absorption coefficient, mm^−1^		0.286
F(000)		568
Crystal size, mm		0.20 × 0.12 × 0.08
Crystal color / shape		Colorless / prism
θ range for data coll.		3.05 to 24.08º
Index range		−10 ≤ *h* ≤11
		−15 ≤ *k* ≤ 15
		−11 ≤ *l* ≤ 11
Completeness, θ = 26.37°		99.6 %
Max. / min. transmission		0.977 / 0.945
Goodness–of–fit on *F*^2^		1.016
Reflec. collect./unique (*R*_int_)		5646/2095 (0.032)
Observed reflections [I > 2σ(I)]		1612
Weights, w		[σ^2^(F_o_^2^) + (0.088P)^2^ + 0.22P]^-1^ P = [Max((F_o_^2^,0) + 2F_c_^2^]/3
Data / restraints / parameters		2095 / 0 / 184
Final *R* indexes^a^ [I > 2σ(I)]		*R_1_* = 0.0474, *wR_2_* = 0.1296
*R* indices (all data)		*R_1_* = 0.0637, *wR_2_* = 0.1479
Extinction coefficient		0.07(1)
Larg. peak & hole, e Å^−3^		0.180 / -0.246

^a^ R indices defined as: *R_1_*=Σ||*F_o_*|-|*F_c_*||/Σ|*F_o_*|, *wR_2_*=[Σ*w*(*F_o_*^2^-*F_c_*^2^)^2^/Σ*w*(*F_o_*^2^)^2^]^1/2^.

The phenyl rings show a delocalized bonding structure, with C-C distances ranging from 1.370(3) to 1.388(3) Å for the chlorine-containing phenyl, where d(C_ph_-Cl) = 1.724(3) Å, and from 1.359(4) to 1.400(3) Å for the phenol ring, where d(C_ph_-OH) = 1.353(3) Å. We found for the carbonyl C(8)=O(2) and C(7)=N(1) groups distances of 1.226(2) and 1.272(3) Å, respectively, as expected for formal double bonds. The N(1)-N(2) length of 1.376(3) Å agrees with the single bond character of this link. The molecule conformation is stabilized by a strong intra-molecular O-H…N bond [d(O(1)···N(1)) = 2.632 Å, ∠(O(1)-H(1)···N(1)) = 145.7°]. As shown in Figure 2 the hydrazone adopts the *E* configuration in the crystal state.

**Table 2:** Bond lengths [Å] and angles [°] for salicylaldehyde 2-chlorobenzoyl hydrazone (H_2_LASSBio-466).

Atoms	Bond lengths (Å)	Atoms	Angles (°)
Cl-C(10)	1.724(3)	O(2)-C(8)-C(9)	123.2(2)
O(1)-C(1)	1.353(3)	N(1)-C(7)-C(2)	121.1(2)
O(2)-C(8)	1.226(3)	N(2)-C(8)-C(9)	114.7(2)
N(1)-N(2)	1.376(3)	N(1)-N(2)-C(8)	118.6(2)
N(1)-C(7)	1.272(3)	N(2)-N(1)-C(7)	117.2(2)
N(2)-C(8)	1.346(3)	C(1)-C(2)-C(3)	118.3(2)
C(1)-C(2)	1.400(3)	C(1)-C(2)-C(7)	122.2(2)
C(1)-C(6)	1.384(4)	C(1)-C(6)-C(5)	120.5(3)
C(2)-C(3)	1.390(3)	C(2)-C(1)-C(6)	119.7(2)
C(2)-C(7)	1.445(3)	C(2)-C(3)-C(4)	121.1(3)
C(3)-C(4)	1.374(4)	C(3)-C(4)-C(5)	119.5(3)
C(4)-C(5)	1.371(5)	C(3)-C(2)-C(7)	119.5(2)
C(5)-C(6)	1.359(4)	C(4)-C(5)-C(6)	120.8(3)
C(8)-C(9)	1.494(3)	C(8)-C(9)-C(10)	122.4(2)
C(9)-C(10)	1.393(3)	C(8)-C(9)-C(14)	119.9(2)
C(9)-C(14)	1.388(3)	C(9)-C(10)-Cl	120.8(2)
C(10)-C(11)	1.381(4)	C(9)-C(10)-C(11)	121.1(3)
C(11)-C(12)	1.376(4)	C(9)-C(14)-C(13)	121.5(3)
C(12)-C(13)	1.371(4)	C(10)-C(9)-C(14)	117.6(2)
C(13)-C(14)	1.370(3)	C(10)-C(11)-C(12)	119.5(3)
		C(11)-C(10)-Cl	118.1(2)
		C(11)-C(12)-C(13)	120.3(3)
		C(12)-C(13)-C(14)	119.9(3)
		O(1)-C(1)-C(2)	122.4(2)
		O(1)-C(1)-C(6)	118.0(2)
		O(2)-C(8)-N(2)	122.1(2)

The solid is further stabilized by a medium-strength inter-molecular N-H···O_carbonyl_ bond [d(N(2)···O(2)) = 2.885 Å, ∠(N(2)-H(2)···O(2’)) = 166.2°] between neighboring molecules, symmetry-related to each other through a glide plane, involving the imine group of a molecule as a donor and the carbonyl oxygen of the other as an acceptor. This gives rise to a polymeric structure that extends along the crystal *c-axis* (see Figure 2).

### 2.3. Spectroscopic Characterization

The vibrations at 3450–3436 cm^−1^ in the infrared spectra of the free acylhydrazones attributed to ν(O-H) disappear in those of the complexes, in accordance with deprotonation of the phenol group [9]. The ν(C=O) absorption at 1657 cm^−1^ in the spectrum of the H_2_LASSBio-466 was not found in the spectrum of the complex **1**, indicating coordination of an enolate oxygen [10,11]. The ν(C=O) absorption at 1676 cm^−1^ in the spectrum of the H_2_LASSBio-1064 shifts to 1618 cm^−1^ in the spectrum of **2**, indicating coordination through the carbonyl oxygen [10,11,12,13]. The vibrations attributed to ν(C=N) at 1625 and 1624 cm^−1^ in the infrared spectra of the hydrazones shift to 1614 and 1611 cm^−1^, respectively, in the spectra of the complexes, in agreement with coordination of the azomethine nitrogen [10,11,12,13]. A new absorption at 1604 cm^−1^ in the spectrum of complex **1** was attributed to the ν(OH_2_) vibration.

The NMR spectra of the acylhydrazones and their zinc(II) complexes were recorded in DMSO-*d*_6_. The ^1^H resonances were assigned on the basis of chemical shifts and multiplicities. The carbon type (C, CH) was determined by using distortionless enhancement by polarization transfer (DEPT 135) experiments. The assignments of the protonated carbons were made by 2D heteronuclear multiple quantum coherence experiments (HMQC).

The signals of all hydrogens and carbons are duplicated in the ^1^H- and ^13^C-NMR spectra of H_2_LASSBio-466, indicating the presence of the *E* and *Z* configurational isomers in the DMSO-*d*_6_ solution. In fact, two N(2)-H signals were observed at δ 11.05 and 9.84 ppm, which were attributed to the *Z* and *E* isomers, respectively. In the first N(2)-H is hydrogen bonded to the phenol oxygen, while in the latter it is hydrogen bonded to the solvent [10,11,12,13]. Only one signal was found for each hydrogen and each carbon in the ^1^H- and ^13^C-NMR spectra of H_2_LASSBio-1064. These signals are compatible with the presence of the *E* configurational isomer.

Only one signal was observed for each hydrogen and each carbon in the spectra of complexes **1**-**2**. The O(1)-H signals were absent in the spectra of all complexes, in agreement with deprotonation and formation of a phenolate group. In the spectrum of **1** the N(2)-H signal disappears, suggesting deprotonation of the hydrazone upon coordination. Hence in **1** a di-anionic hydrazone ligand is attached to the zinc(II) center. In the spectrum of **2** the N(2)-H signal was observed, according with the presence of a mono-anionic hydrazone ligand. In the ^1^H-NMR spectra of the complexes the signals of all hydrogens undergo significant shifts in relation to their positions in the free hydrazones. Similarly, the signals of C=N, C=O and the phenol carbons undergo significant shifts in complexes **1** and **2**, indicating coordination through the O_phenol_-N-O chelating system. Hence in **1**-**2** the hydrazones adopt the *E* configuration. 

### 2.4. Anti-Nociceptive Activity of H_2_LASSBio-466, H_2_LASSBio-1064, [Zn(LASSBio-466)H_2_O]_2_ (***1***) and [Zn(HLASSBio-1064)Cl]_2_ (***2***)

The anti-nociceptive profiles of the free ligands H_2_LASSBio-466, H_2_LASSBio-1064 and their zinc(II) complexes **1** and **2** were evaluated using three well-accepted pain models, namely acetic acid-induced writhing, formalin-induced nociception and hot plate test. The acetic acid-induced abdominal writhing and hot-plate test have been reported to be useful to investigate peripheral and central activity, respectively, while the formalin-induced nociception is valuable to detect both effects, including inflammatory pain.

All compounds were evaluated at a dose of 100 μmol/kg (p.o). Indomethacin, a COX-1 selective inhibitor (100 μmol·kg^−1^, p.o.) and dipyrone, a COX-3 selective inhibitor (100 μmol·kg^−1^, p.o.) were used as standard drugs in the peripheral nociception models, while morphine (15 μmol·kg^−1^, i.p.) was used as standard in the hot-plate test. The analgesic activity of H_2_LASSBio-466, its regioisomer H_2_LASSBio-1064, and their complexes [Zn(LASSBio-466)H_2_O]_2_ (**1**) and [Zn(HLASSBio-1064)Cl]_2_ (**2**) was initially evaluated employing the acetic acid-induced abdominal writhing model in mice and compared with those of the standards [14]. 

As shown in Table 3, all compounds produced marked inhibition of acetic acid-induced writhing response. However, the anti-nociceptive activity appears to have been favored by complex formation in the case of complex **1**, given its increased activity compared to the H_2_LASSBio-466 free ligand. 

**Table 3:** Effect of H_2_LASSBio-466, its regioisomer H_2_LASSBio-1064, their zinc(II) complexes, indomethacin and dipyrone (100 µmol·kg^−1^, p.o.) on the 0.6% acetic acid-induced abdominal constrictions in mice, for a period of 25 min.

Substance	n	Writhing number Mean ± S.E.M.	% of inhibition Mean ± S.E.M.
Control	6	37.5 ± 1.4	___
Indomethacin	6	6.0 ± 3.1 **	84.0 ± 8.3 **
Dipyrone	6	8.3 2.7 **	77.8 ± 7.2 **
H_2_LASSBio-466	6	14.8 ± 2.2 **	60.4 ± 6.0 **
H_2_LASSBio-1064	6	7.0 ± 1.1 **	81.3 ± 3.0 **
[Zn(LASSBio-466)H_2_O]_2_ (**1**)	6	6.6 ± 1.4 **	82.3 ± 3.7 **
[Zn(HLASSBio-1064)Cl]_2_ (**2**)	6	10.8 ± 2.3 **	70.9 ± 6.2 **

Data are expressed as mean ± S.E.M. Statistical differences between the treated and the control groups were evaluated by ANOVA and Dunnett tests and the asterisks denote the levels of significance in comparison with control groups, ** P < 0.01.

The neurogenic and inflammatory pain was evaluated using the formalin test and analyzing the first and the second phases of the nociceptive response, respectively [15]. In this model, H_2_LASSBio-466 was effective in inhibiting only the first phase, while [Zn(LASSBio-466)H_2_O]_2_ (**1**), like indomethacin, was active in the second phase, indicating its ability to inhibit nociception associated with inflammatory response (Table 4). H_2_LASSBio-1064 was able to inhibit both neurogenic and inflammatory phases anticipating a distinct pharmacological profile. However this effect decreases on coordination to zinc(II) in complex **2**. These results suggest that coordination seems to be a good strategy to improve the antinociception profile of the prototype H_2_LASSBio-466 associated with an inflammatory pain.

**Table 4:** Effect of prototypes H_2_LASSBio-466, H_2_LASSBio-1064, their zinc(II) complexes and indomethacin (100 µmol·kg^−1^, p.o.) on formalin (2.5%) test in mice.

Substance	n	Phase 1 Mean ± S.E.M.	Phase 2 Mean ± S.E.M.	% of inhibition (Mean ± S.E.M.)	
				Phase 1	Phase 2
Control	5	54.8 ± 2,3	227.6 ± 22.7	—	—
Indomethacin	5	57.1 ± 8.5	115.9 ± 3.3 *	0	49.1 ± 1.4 *
H_2_LASSBio-466	5	29.3 ± 8.3 **	182.4 ± 17.4	46.5 ± 8.3 **	19.8 ± 9.7
H_2_LASSBio-1064	5	25.7 ± 4.9 *	117.0 ± 19.1 *	53.1 ± 8.9 *	48.5 ± 8.4 *
[Zn(LASSBio-466)H_2_O]_2_ (**1**)	5	40.6 ± 12.7	142.8 ± 23.1 *	25.9 ± 15.7	37.3 ± 10.1 *
[Zn(HLASSBio-1064)Cl]_2_ (**2**)	5	39.6 ± 7.8	161.3 ± 36.0	27.7 ± 13.1	29.2 ± 13.9

Data are expressed as mean ± S.E.M. Statistical differences between the treated and the control groups were evaluated by test t and Mann-Whitney tests and the asterisks denote the levels of significance in comparison with control groups, * P < 0.05 and ** P < 0.01.

In order to investigate an occasional central anti-nociceptive activity for prototypes H_2_LASSBio-466, H_2_LASSBio-1064 and their zinc(II) complexes, the studied compounds were evaluated in the hot plate test using morphine (15 µmol·kg^−1^, i.p.) as standard [16]. As shown in Table 5, these compounds did not increase the latency of response significantly, showing that they do not present activity in the supra-spinal analgesia, while morphine induced a marked increase in the latency of the animals at 60 min (9.0 ± 1.6 s), 90 min (7.4 ± 0.8 s), 120 min (5.3 ± 0.8 s) and 150 min (2.5 ± 0.2 s). These results indicate that the studied compounds do not have any central anti-nociceptive activity.

**Table 5:** Time course effect of prototypes H_2_LASSBio-466, H_2_LASSBio-1064, their zinc(II) complexes (100 µmol·kg^−1^, p.o.) and morphine (15 µmol·kg^−1^, i.p.) on hot-plate test in mice.

Substance	n	Mean ± S.E.M.				
		0 min	60 min	90 min	120 min	150 min
Control	6	2.8 ± 0.2	2.9 ± 0.3	1.6 ± 0.2	2.3 ± 0.40	1.7 ± 0.1
Morphine	6	1.8 ± 0.5	9.0 ± 1.6 *	7.4 ± 0.8 *	5.3 ± 0.8 *	2.5 ± 0.2
H_2_LASSBio-466	6	2.1 ± 0.21	2.3 ± 0.4	2.1 ± 0.2	2.2 ± 0.2	2.6 ± 0.1
H_2_LASSBio-1064	6	1.7 ± 0.18	2.6 ± 0.2	2.1 ± 0.2	2.5 ± 0.3	3.5 ± 0.3
[Zn(LASSBio-466)H_2_O]_2_ (**1**)	6	2.1 ± 0.2	4.2 ± 0.8	2.4 ± 0.6	3.6 ± 0.6	5.4 ± 1.2
[Zn(HLASSBio-1064)Cl]_2_ (**2**)	6	1.5 ± 0.1	2.0 ± 0.5	2.3 ± 0.5	2.9 ± 1.0	1.9 ± 0.2

Data are expressed as mean ± S.E.M. Statistical differences between the treated and the control groups were evaluated by ANOVA and Dunnett tests and the asterisks denote the levels of significance in comparison with control groups, * P < 0.05.

To better assess the potential anti-inflammatory activity of the free acylhydrazones and their zinc(II) complexes, the zymosan-induced peritonitis assay was performed [17]. As seen in Table 6, all compounds showed some level of inhibition in this cell migration model comparable or superior to indomethacin.

**Table 6:** Effect of prototypes H_2_LASSBio-466, H_2_LASSBio-1064, their zinc(II) complexes and indomethacin (100 µmol·kg^−1^, p.o.) on the zymosan-induced peritonitis in mice.

Substance	n	Cell Number × 10^6^/mL Mean ± S.E.M.	% of inhibition Mean ± S.E.M.
Control	7	38.0 ± 1.0	___
Saline	7	5.0 ± 0.8	___
Indomethacin	7	17.7± 1.0 **	53.4 ± 2.7 **
H_2_LASSBio-466	7	11.4 ± 1.4 **	70.0 ± 3.8 **
H_2_LASSBio-1064	7	8.4 ± 0.9 **	77.8 ± 2.4 **
[Zn(LASSBio-466)H_2_O]_2_ (**1**)	7	10.7 ± 1.8 **	71.8 ± 4.9 **
[Zn(HLASSBio-1064)Cl]_2_ (**2**)	7	13.4 ± 1.5 **	64.7 ± 4.0 **

Data are expressed as mean ± S.E.M. Statistical differences between the treated and the control groups were evaluated by ANOVA and Dunnett tests and the asterisks denote the levels of significance in comparison with control groups, ** P < 0.01.

In fact, H_2_LASSBio-466, H_2_LASSBio-1064, [Zn(LASSBio-466)H_2_O]_2_ and [Zn(HLASSBio-1064)Cl]_2_, presented 70.0%, 77.8%, 71.8% and 64.7% of inhibition, respectively, while indomethacin inhibited cell-migration by 53.4%.

## 3. Experimental

### 3.1. General

All common chemicals were purchased from Aldrich and used without further purification. Partial elemental analyses were performed on a Perkin Elmer CHN 2400 analyzer. A YSI model 31 conductivity bridge was employed for molar conductivity measurements (1 × 10^−3^ mol L^−1^, DMF). Infrared spectra (4000-400 cm^-1^) were recorded on a Perkin Elmer FT-IR Spectrum GX spectrometer using KBr plates. NMR spectra were obtained with a Bruker DPX-200 Avance (200 MHz) spectrometer using DMSO-*d*_6_ as the solvent and TMS as internal reference.

### 3.2. Synthesis of H_2_LASSBio-466 and H_2_LASSBio-1064

The synthesis of H_2_LASSBio-466 and H_2_LASSBio-1064 were performed using a previously described methodology [18]. Briefly, salicylaldehyde (2-hydroxybenzaldehyde) was added to a solution of 2-chlorobenzohydrazide or 4-chlorobenzohydrazide (an equimolar amount) in absolute ethanol containing one drop of 37% hydrochloric acid. The mixture was stirred at room temperature for 2 hours until extensive precipitation was observed. Next, the solvent was partially concentrated at reduced pressure and the resulting mixture was poured into cold water. The precipitate formed was filtered out and dried under vacuum producing the desired (*E,Z*)-salicylaldehyde 2-chlorobenzoyl hydrazone (H_2_LASSBio-466) and (*E*)-salicylaldehyde 4-chlorobenzoyl hydrazone (H_2_LASSBio-1064) in 87% and 91% yield, respectively. The melting point and ^1^H-NMR data for both compounds were in agreement with previous reports [19,20].

### 3.3. Synthesis of Zinc(II) Complexes

#### 3.3.1. Synthesis of [Zn(LASSBio-466)H_2_O]_2_ (**1**) and [Zn(HLASSBio-1064)Cl]_2_ (**2**)

Complexes **1** and **2** were obtained by mixing an ethanol solution of the desired hydrazone with zinc chloride and triethylamine in 1:1:1 ligand-to-metal-to-triethylamine molar ratio. The resulting solids were washed with ethanol followed by diethylether and then dried *in vacuo*. 

*[Zn(LASSBio-466)H_2_O]_2_* (**1**). Yield 68%.Yellow solid. Anal. Calc. for C_28_H_22_N_4_O_6_Cl_2_Zn_2_ (712.18): C, 47.26%; H, 2.85%; N, 7.40%. Found: C, 47.22%; H, 3.11%; N, 7.87%. M.P. > 300 °C. Molar conductivity: 8.23 Ω^−1^cm^2^ mol^−1^. IR: ν(C=N) 1614, ν(Zn-OH_2_) 1604, ν(phenolic, CO) 1329. ^1^H-NMR: δ (ppm) = 7.54 (1H, H7), 7.47–7.59 (1H, H14), 7.52 (1H, H11), 7.51 (1H, H5), 7.44–7.47 (1H, H3), 7.32 (1H, H13), 7.28 (1H, H13), 6.79 (1H, H6), 6.70 (1H, H4). ^13^C-NMR: δ (ppm) = 169.73 (C8), 158.02 (C1), 150.59(C7), 135.87 (C10), 135.09 (C12), 134.37 (C13), 131.38 (C5), 130.35 (C11), 129.81 (C14), 127.48 (C3), 119.69 (C9, C2), 119.35 (C4), 118.37 (C6). 

*[Zn(HLASSBio-1064)Cl]_2_* (**2**). Yield 71%. Yellow solid. Anal. Calc. for C_28_H_20_N_4_O_4_Cl_4_Zn_2_ (749.11): C, 44.72%; H, 2.24%; N, 7.10%. Found: C, 44.89%; H,2.69%; N, 7.48%. M.P. > 300 °C. Molar conductivity: 11.35 Ω^−1^cm^2^ mol^−1^. IR: ν(N-H) 3179, ν(C=O) 1618, ν(C=N) 1611, ν(phenolic, CO) 1281. ^1^H-NMR: δ (ppm) = 8.99 (1H, H7), 8.01 (2H, H11,H13), 7.59 (2H, H10,H14), 7.38 (1H, H3), 7.24 (1H, H5), 6.73 (1H, H6), 6.89 (1H, H4). ^13^C-NMR: δ (ppm) = 168.88 (C8), 166.81 (C1), 155.61 (C7), 136.83 (C12), 131.62 (C5), 129.68 (C10,C14), 129.58 (C3), 128.79 (C11, C13), 119.53 (C4), 118.75 (C9, C2), 116.54 (C6). 

### 3.4. X-ray Crystallography

The X-ray diffraction measurements were performed on an Enraf-Nonius Kappa-CCD diffractometer with graphite-monochromated MoKα (λ = 0.71073 Å) radiation. Diffraction data were collected (φ and ω scans with κ-offsets) with COLLECT [21]. Integration and scaling of the reflections were performed with HKL DENZO-SCALEPACK [22] suite of programs. The unit cell parameters were obtained by least-squares refinement based on the angular settings for all collected reflections using HKL SCALEPACK. The structure was solved by direct methods with SHELXS-97 [23] and the molecular model refined by full-matrix least-squares procedure on *F^2^* with SHELXL-97 [24]. The hydrogen atoms were included in the molecular model at stereo-chemical positions and refined with the riding model.

### 3.5. Anti-nociceptive and anti-inflammatory activities of H_2_LASSBio-466, H_2_LASSBio-1064, [Zn(LassBio-466)H_2_O]_2_ (***1***) and [Zn(HLassBio-1064)Cl]_2_ (***2***)

#### 3.5.1. Animals

Experiments were conducted using Swiss mice obtained from the BIOCEN - UFAL breeding unit, weighing 20–30 g each, males or females, adult, with 6 to 8 weeks of age, distributed in groups of up to 6–8 animals for treatment. The animals were maintained with free access to food and water and kept at 25–28 °C under a controlled 12 h light·dark^−1^ cycle. All animals were manipulated according to the norms established by the Ethics Commission - UFAL for handling animals (protocol number: 026681/2009-23).

#### 3.5.2. Writhing Test

This test was performed as described by Collier and coworkers [14]. Acetic acid (0.6%, v/v) was administered i.p. in a volume of 0.1 mL·10 g^−1^. The number of writhes, a response consisting of contraction of an abdominal wall, pelvic rotation followed by hind limb extension, was counted during continuous observation for 20 min. beginning 5 min. after the acetic acid injection. The prototypes H_2_LASSBio-466, H_2_LASSBio-1064 and their zinc(II) complexes, indomethacin and dipyrone were administered at the dose of 100 µmol·kg^−1^ (p.o), 40 min. before the acetic acid injection. Control group received 10 mL·kg^−1^ of vehicle (arabic gum, p.o.). Anti-nociceptive activity was expressed as inhibition percentage of the usual number of writhing observed in control animals.

#### 3.5.3. Formalin-Induced Pain in Mice

The formalin test was performed as described by Hunskaar and Hole [15]. Animals received 20 µL of a 2.5% formalin solution (0.92% formaldehyde in saline) in the ventral surface of the right hind paw. They were observed from 0 to 5 min. (neurogenic phase) and from 15 to 30 min. (inflammatory phase) after injection and the time they spent licking the injected paw was recorded and considered as indicative of nociception. The prototypes H_2_LASSBio-466, H_2_LASSBio-1064, their zinc(II) complexes and indomethacin were administered at the dose of 100 µmol·kg^−1^ (p.o), 40 min. before formalin injection. Control group received 10 mL·kg^−1^ of vehicle (arabic gum, p.o.).

#### 3.5.4. Hot-Plate Test

Mice were treated according to the method described by Eddy and Leimbach [16]. Each mouse was placed on the hot plate set at 54 ± 1.0 °C and the time of paw licking was recorded before and 30 min. after oral administration of the tested compounds. The prototypes H_2_LASSBio-466, H_2_LASSBio-1064 and their zinc(II) complexes were administered at the dose of 100 µmol·kg^−1^ (p.o). Control group received 10 mL·kg^−1^ of vehicle (arabic gum, p.o.). Morphine was also used as a drug standard at the dose of 15 μmol·kg^−1^ (i.p.). Analgesia was defined as an increase in the latency of paw licking, and the latency times were compared with the values obtained for control. Sixty seconds were taken as the cut-off time to avoid mouse tissue damage.

#### 3.5.5. Zymosan-Induced Peritonitis

Peritoneal inflammation was induced according to the method described by Doherty [17]. A solution of zymosan A (Sigma-Aldrich, 2 mg·mL^−1^) was prepared in saline (0.9% NaCl) and injected into the peritoneal cavity of mice (0.5 mL). Six hours after injection of zymosan A, the animals were sacrificed by cervical dislocation and the peritoneal cavity was washed with cold Hank’s solution (3 mL). The prototypes H_2_LASSBio-466, H_2_LASSBio-1064, their zinc(II) complexes and indomethacin were administered at the dose of 100 µmol·kg^−1^ (p.o), 40 min. before zymosan A injection. The control group received 10 mL·kg^−1^ of vehicle (arabic gum, p.o.). The number of cells was quantified using an optical microscope, and a 100 x lens.

#### 3.5.6. Statistical Analysis

Data obtained from animal experiments are represented by mean ± standard error of the mean (Mean ± S.E.M.). Statistical differences between the treated and the control groups were evaluated by test t of Student or ANOVA in the tutorial Prisma®. Values were considered significant if * P < 0.05, ** P < 0.01 and *** P < 0.001.

## 4. Conclusions

The studied compounds, evaluated at a dose of 100 μmol/kg (p.o), showed marked inhibition of acetic acid-induced writhing response, with the anti-nociceptive activity being favored by zinc(II) complex formation in the case of complex **1**. The compounds also have antinociception profile associated with inflammatory pain, with no activity in murine analgesic model of central pain. In the formalin model, H_2_LASSBio-466 was effective in inhibiting only the first phase, while its zinc(II) complex **1**, like indomethacin, was active in the second phase, indicating its ability to inhibit nociception associated with inflammatory response. Hence coordination to zinc(II) altered the pharmacological profile of H_2_LASSBio-466. Moreover, the salicyladehyde *N*-acylhydrazone derivatives and their zinc(II) complexes showed comparable or superior inhibition of cell-migration process to indomethacin, indicating an expressive anti-inflammatory profile.

The different activities of the complexes could be explained by their releasing hydrazone at different rates during the assays. In addition, complex **1** contains a di-anionic hydrazone while **2** contains a mono-anionic hydrazone. Hence different electronic effects could also influence the distinct pharmacological profiles of complexes **1** and **2**. In addition, **1** contains a water molecule in the metal coordination sphere while **2** contains a chloride ion*.* Finally, coordination could in principle alter the bioavailability of the two hydrazones in different manners. The above-mentioned results suggest that further studies on the anti-inflammatory properties of this class of compounds and their zinc(II) complexes should be carried out in order to investigate their mechanism of action

## Supplementary Material

CCDC 805145 contains the supplementary crystallographic data for salicylaldehyde 2-chlorobenzoyl hydrazone (H_2_Lassbio-466). This data can be obtained free of charge via http://www.ccdc.cam.ac.uk/conts/retrieving.html, or from the Cambridge Crystallographic Data Centre, 12 Union Road, Cambridge CB2 1EZ, UK; fax: (+44) 1223-336-033; or Email: deposit@ccdc.cam.ac.uk.
